# Succession of endophytic fungi and rhizosphere soil fungi and their correlation with secondary metabolites in *Fagopyrum dibotrys*

**DOI:** 10.3389/fmicb.2023.1220431

**Published:** 2023-08-01

**Authors:** Nan Ma, Dengpan Yin, Ying Liu, Ziyong Gao, Yu Cao, Tongtong Chen, Ziyi Huang, Qiaojun Jia, Dekai Wang

**Affiliations:** Key Laboratory of Plant Secondary Metabolism Regulation in Zhejiang Province, College of Life Sciences and Medicine, Zhejiang Sci-Tech University, Hangzhou, Zhejiang, China

**Keywords:** endophytic fungi, rhizosphere soil fungi, *Fagopyrum dibotrys*, secondary metabolites, medicinal plants

## Abstract

Golden buckwheat (*Fagopyrum dibotrys*, also known as *F. acutatum*) is a traditional edible herbal medicinal plant with a large number of secondary metabolites and is considered to be a source of therapeutic compounds. Different ecological environments have a significant impact on their compound content and medicinal effects. However, little is known about the interactions between soil physicochemical properties, the rhizosphere, endophytic fungal communities, and secondary metabolites in *F. dibotrys*. In this study, the rhizosphere soil and endophytic fungal communities of *F. dibotrys* in five different ecological regions in China were identified based on high-throughput sequencing methods. The correlations between soil physicochemical properties, active components (total saponins, total flavonoids, proanthocyanidin, and epicatechin), and endophytic and rhizosphere soil fungi of *F. dibotrys* were analyzed. The results showed that soil pH, soil N, OM, and P were significantly correlated with the active components of *F. dibotrys*. Among them, epicatechin, proanthocyanidin, and total saponins were significantly positively correlated with soil pH, while proanthocyanidin content was significantly positively correlated with STN, SAN, and OM in soil, and total flavone content was significantly positively correlated with P in soil. In soil microbes, *Mortierella, Trechispora, Exophiala*, Ascomycota_unclassified, *Auricularia, Plectosphaerella, Mycena*, Fungi_unclassified, Agaricomycetes_unclassified, *Coprinellus*, and *Pseudaleuria* were significantly related to key secondary metabolites of *F. dibotrys*. *Diaporthe* and Meripilaceae_unclassified were significantly related to key secondary metabolites in the rhizome. This study presents a new opportunity to deeply understand soil-plant-fungal symbioses and secondary metabolites in *F. dibotrys*, as well as provides a scientific basis for using biological fertilization strategies to improve the quality of *F. dibotrys*.

## Introduction

Golden buckwheat (*Fagopyrum dibotrys*, also known as *F. acutatum*) belongs to the dicotyledonaceae family Polygonaceae, which is widely distributed throughout the temperate North Zone and mainly grown in China and Southeast Asia (Jing et al., 2016; Zhang et al., 2021a; He et al., 2022). As a traditional edible herbal medicinal plant with important nutritional and economic values, the rhizome of *F. dibotrys* harbors over 100 types of secondary metabolites, such as flavonoids, saponins, phenolics, triterpenoids, proanthocyanidins, and epicatechin (Wang et al., 2005). These secondary metabolites exhibited valuable pharmacological and physiological activities, such as antioxidant, antipyretic, antibacterial, anti-inflammatory, and antitumor effects (Chen and Li, 2016; Li et al., 2021). Among the numerous secondary metabolites, flavonoids are the main active medicinal ingredient in *F. dibotrys*. Epicatechin, as a marker for evaluating the quality of *F. dibotrys* in the Chinese Pharmacopeia (2020 edition), has strong antioxidant and tumor cell growth inhibitory activities and plays an important role in the regulation of human blood vessels (Sinegre et al., 2019; Gu et al., 2022). Proanthocyanidins are natural glycosidase inhibitors formed by epicatechin polymerization that have strong antioxidant and hypoglycemic effects (Li et al., 2021). Saponins are compounds composed of carbohydrates and triterpenes or steroidal aglycones, which have anti-inflammatory, antioxidant, nervous system protection, and other pharmacological effects (Juang and Liang, 2020). In addition, the stems and leaves of *F. dibotrys* also contain abundant secondary metabolites. Therefore, *F. dibotrys* is also widely used in health care products, feed, vegetables, and feed resources. For example, the grains of *F. dibotrys* are commonly used in making foods such as flour, tea, and side dishes. Owing to its significant health benefits and pharmacological effects, buckwheat has been approved as a functional food in China (Chen and Li, 2016). However, owing to its weak reproductive capacity and excessive human exploitation, the wild *F. dibotrys* resource has rapidly depleted and is now listed as a Class 2 endangered wild plant in China (Wang et al., 2018).

The bioactive ingredients (secondary metabolites) of medicinal plants are influenced by several external environmental factors such as soil pH, soil nutrients, temperature, humidity, and the microbial community of the soil rhizosphere (Sampaio et al., 2016; Zhai et al., 2017; Li et al., 2020). Rhizosphere soil and endophytic fungi affect plant growth, development, physiology, and metabolism in different ways. For example, soil rhizosphere fungi received photosynthates from host plants and, in turn, helped host plants increase their absorption of nutrients (nitrogen and phosphorus), thus affecting the secondary metabolism of host plants and ecological functions (Lau and Lennon, 2011; Bennett et al., 2017; Jiang et al., 2017). Regarding various environmental conditions, soil is the most important factor in stimulating secondary metabolites, as it controls the movement and availability of water, air, and nutrients (Atyane et al., 2016; Yang et al., 2018; Xiao et al., 2022). Several studies have shown that soil type and cultivar influence the available nitrogen, phosphorus, potassium, and organic carbon content of rhizosphere soil, thereby affecting rhizosphere microbial communities, plant growth, and secondary metabolites (Sampaio et al., 2016; Ren et al., 2022; Hou et al., 2023). Organic matter (OM) can promote the formation of soil structure, improve the fertilizer holding capacity and buffer capacity of the soil, promote the growth and development of plants, and affect secondary metabolites (Witzgall et al., 2021). It has been reported that OM improves the biosynthesis of secondary metabolites in many medicinal plants by enhancing the nutritional availability and cation exchange capacity of essential elements in soil, such as marigold (Pandey et al., 2015), *Sinopodophyllum hexandrum* (Liu et al., 2018), and saffron (Chaouqi et al., 2023). The soil pH value impacts the soil microbial community and secondary metabolites by affecting the concentration of various ions in the soil (Hassan and Mathesius, 2012; Naz et al., 2022). Within the pH range suitable for plant growth, the pH value has a significant impact on the accumulation of plant secondary metabolites. For example, the *cis*-3-Gg content of saffron in moderately alkaline pH is slightly higher than that in neutral or weakly alkaline pH (Kandimalla et al., 2020). It has been reported that the soil pH had a positive effect on the total sesquiterpene lactone **(**SL) content in *Arnica montana* (Greinwald et al., 2022). In addition to these external environmental factors, endophytic fungi, an important component in plant tissues and intercellular spaces, play important roles in the growth, development, ecological adaptation, and fitness benefits of host plants (Rodriguez et al., 2009). Endophytic fungi are a group of host-associated fungal communities that reside inside plant tissues by establishing a mutually beneficial plant micro-ecosystem with their host plant without causing disease symptoms or adverse effects (Radic and Strukelj, 2012; Deshmukh et al., 2015, 2022). In general, host plants provide carbohydrates that are critical to the habitat and growth of endophytic fungi. In return, a secondary metabolite produced by endophytic fungi can be provided to plants to help them withstand various pressure sources, such as drought, damage, salinity increase, and nutrition imitation (Deshmukh et al., 2015; Liu et al., 2015; Alam et al., 2021). Endophytic fungi have a positive impact on the physiological activity of host plants in various ways, such as producing hormones (indoleacetic acid, etc.), biosynthesizing and acquiring nutrients, secreting stress-adaptor metabolites, thus promoting plant growth and development, and resisting biological and abiotic stress (Igiehon et al., 2021; Poveda et al., 2021; Watts et al., 2023). In addition, endophytic fungi have attracted great attention, as they not only provide beneficial effects for host plants but have also been identified as possible sources of novel secondary metabolites with high therapeutic value, including antioxidant, anticarcinogenic, antibacterial, and antiviral molecules (Gouda et al., 2016; Uzma et al., 2018; Devi et al., 2023).

In recent years, numerous studies have shown that the plant contains a high number of endophytic fungi using high-throughput sequencing technology (Torres and Kelley, 2018). Many reports indicate a significant correlation between the endophytic fungal community and the content of secondary metabolites in medicinal plants from different areas. For example, the accumulation of secondary metabolites in *Cynomorium songaricum* is closely related to the composition of the endophytic fungal community (Cui et al., 2018, 2019). Chen et al. (2021) showed that five secondary metabolites of *Rheum palmatum* from different production areas in Gansu were positively correlated with the diversity and abundance of endophytic fungi (Chen et al., 2021). Dang et al. (2021) reported that the contents of three secondary metabolites of licorice roots increased per year and that the endophytic fungal communities were more sensitive to secondary metabolites than the AMF communities (Dang et al., 2021). As a perennial plant, there must be different secondary metabolites in *F. dibotrys* from different areas, according to traditional experience and Chinese pharmacopeia. However, to date, little is known about the comprehensively assessed secondary metabolites, diversity, community composition, and distribution patterns of soil rhizosphere fungi and endophytic fungi, as well as the correlations between them in *F. dibotrys* from different areas.

In this study, the content of bioactive secondary metabolites housed within the *F. dibotrys* rhizome from five areas was determined using spectrophotometry and HPLC. The ITS region of fungal riboRNA (rRNA) genes from five different areas of *F. dibotrys* was sequenced using the high-throughput sequencing method. Then, the diversity and composition of the fungal community in the soil and rhizome of *F. dibotrys* were analyzed. Moreover, the correlation between soil rhizosphere fungi, endophytic fungi, and the bioactive compounds of the host was also performed. Our results will provide novel information on how rhizosphere fungi and endophytic fungi are involved in the secondary metabolism of *F. dibotry* and facilitate efficient methods to improve the quality of *F. dibotry*.

## Materials and methods

### Sample collection

**Figure 1:**
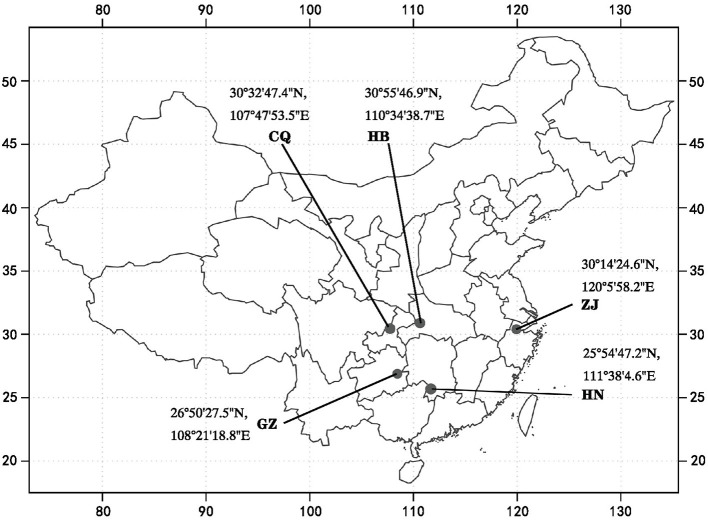
Distribution map of the sampling plot of *F. dibotrys*. CQ, HN, GZ, ZJ, and HB represent Chongqing City, Hunan Province, Guizhou Province, Zhejiang Province, and Hubei Province, respectively.

The wild populations of *F. dibotrys* materials were derived from five different areas of China (Figure 1). Samples were identified as wild *F. dibotrys*. The rhizomes and rhizosphere soil of *F. dibotrys* were sampled in December 2021 from five areas, and staggered sampling was implemented to maintain sample uniformity. Three homogeneous composite samples, set as three replicates, were collected from 'Z'-shaped sampling sites, and each sample consisted of three randomly selected rhizomes and rhizosphere soils of individuals from well-grown *F. dibotrys* plants. Rhizosphere soil (0–40 cm) was collected from rhizomes that cannot be shaken off. A total of 15 rhizomes and 15 soil samples from the rhizosphere soil were taken to the laboratory with an icebox for further treatments. Three soil samples for each individual were thoroughly mixed to obtain a uniform composite soil sample and then divided into two parts, where one part was stale quickly using liquid nitrogen for subsequent DNA extraction, and the other was used to analyze soil physicochemical properties. The rhizomes of *F. dibotrys* were continuously washed with tap water until no soil or impurities could be observed. The rinsed rhizomes were then dried with sterile filter paper. Each rhizome sample was equally divided into two parts: one part was used to determine its content of secondary metabolites, and another part was used to extract DNA. All rhizome samples from different areas were sterilized as previously described (Liu et al., 2018). The rhizomes were sterilized in a refrigerator at −80°C for DNA extraction. The samples were marked as follows: Zhejiang rhizomes (ZJC_1, ZJC_2, and ZJC_3), rhizosphere soil (ZJRh_1, ZJRh_2, and ZJRh_3); Chongqing rhizomes (CQC_1, CQC_2, and CQC_3), rhizosphere soil (CQRh_1, CQRh_2, and CQRh_3); Hubei rhizomes (HBC_1, HBC_2, and HBC_3), rhizosphere soil (HBRh_1, HBRh_2, HBRh_3); Guizhou rhizomes (GZC_1, GZC_2, and GZC_3), rhizosphere soil (GZRh_1, GZRh_2, and GZRh_3); Hunan rhizomes (HNC_1, HNC_2, and HNC_3), and rhizosphere soil (HNRh_1, HNRh_2, and HNRh_3).

### Determination of physicochemical properties in the soil of the rhizosphere

The naturally dried rhizosphere soil was passed through a 2-mm sieve and used to determine its physical and chemical properties. The physicochemical properties of the rhizosphere soil were determined according to the China National Standard Methods. A total of 3.0 g of soil sample was weighed and determined with the soil suspension (1:5 water to soil ratio) using a pH meter (HJ 962-2018). In detail, a total of 0.4 g of soil sample was weighed, and soil total nitrogen (STN) was measured using the Kjeldahl method (sulfuric acid digestion method) (NY/T 1121.24-201). A total of 0.20 g of soil sample was weighted, and soil total phosphorus (STP) was determined using NaOH-molybdenum-antimony anti-spectrophotometry (NY/T 88-1988). A total of 0.2 g of soil sample was weighed, and soil total potassium (STK) was determined using the NaOH fusion method (NY/T 87–1988). A total of 2.5 g of soil sample was weighed, treated with sodium hydroxide, and titrated with acid (alkali-hydrolytic diffusion method) to determine the soil available nitrogen (SAN) (LY/T 1228-2015). A total of 2.5 g of soil sample was weighed, the available phosphorus was extracted using the molybdenum-antimony resistance colorimetric method, and the amount of soil available phosphorus (SAP) was determined using a spectrophotometer (NY/T 1121.7-2014). A total of 2.5 g of soil sample was weighed, and the soil potassium available in the SAK was determined using the ammonium acetate extraction method (NY/T 889–2004). A total of 0.1 g of soil was weighed, and soil organic matter (OM) was determined using the potassium dichromate volumetric method (NY/T 1121.6–2006).

### Measurement of secondary metabolites in the rhizome

The rhizome of *F. dibotrys* was washed, cut, and dried at 60 °C until it was completely dry before being crushed and passed through the 60 mesh sieves. The total flavonoid content of the rhizome was determined using the aluminum trichloride method (Cheng et al., 2019). Briefly, 0.2 g of rhizome powder samples were weighed and placed in a 150-mL triangular flask. Then, 30 mL of a 70% methanol solution was added to the flask. The flask was placed in a thermostatic water shaker set at 65°C and shaken for 2 h (160 r/min). After being filtered, the mixture was filtered, and the resulting filtrate was placed in a 50-mL flask. Methanol solution (70%) was added to bring the solution to the desired volume. Subsequently, 10 mL of the test solution was transferred to a 10-mL volumetric flask. To this flask, 2 ml of aluminum trichloride (0.1 mol/L) and 3 mL of potassium acetate (1 mol/L) solutions were added. The volume was adjusted to the desired level using methanol solution (70%), and the flask was left at room temperature for 30 min. The absorbance value at 420 nm was measured using an enzyme labeling instrument (iMark, Bio-Rad Co., Ltd., CA, USA). The reference standard curve for this analysis was prepared using rutin (purchased from Chengdu Desite Biotechnology Co. Ltd., Chengdu, China). The standard curve is linear in the range of 0–0.10 mg/mL (Y = 1.46X+ 0.0505, R^2^ = 0.9966). The proanthocyanidin content was measured according to the previously described method (Shi et al., 2021). A total of 1.0 g of rhizome powder samples was weighed and added to 25 ml of extraction solvent (materials-to-liquid ratio of 1:25) before being subjected to ultrasonics (1 h). After filtration, the combined filtrates were distilled under a vacuum and concentrated to 2.5 ml of extract. A total of 1.0 ml of concentrate was pipetted into a 10-ml flask and filled the volume; then, 1.0 ml of test solution was taken to determine the proanthocyanidin content. Then, 2.5 ml of 3% vanillin-methanol solution and 2.5 ml of 30% sulfuric acid-methanol solution were added to each test tube and shaken well. The reaction mixture was incubated in a water bath at 30 ° C for 20 min, and the absorbance was measured at 546 nm using an ultraviolet spectrophotometer (Shanghai Yidian Sciences Instrument Co., Ltd., China). The standard curve of the standard proanthocyanidin B2 product (purchased from Chengdu Desite Biotechnology Co. Ltd., Chengdu, China) was used as a reference. The standard curve is linear in the range of 0–0.10 mg/mL (Y = 0.0058X + 0.0067, R^2^ = 0.999). The content of total saponins was also measured based on the previously described method with some modifications (Han et al., 2019). In brief, a total of 1.0 g of rhizome powder samples were extracted by ethanol with an ultrasonic filter, and 0.2 mL of the sample was used for water bath drying. Then, the residue was mixed with 0.2 mL of 5% (w/v) Vanillin in 17 M acetic acid and 0.8 mL of 72% Perchloric acid and reacted in a water bath at 70°C for 10 min. Then, the cooled mixture was mixed with 5 mL of 17 M acetic acid and incubated at room temperature for another 10 min. The absorbance was detected at 550 nm using an ultraviolet spectrophotometer (Shanghai Exact Sciences Instrument Co., Ltd., China), and the standard curve of the standard oleanolic acid product (purchased from Chengdu Desite Biotechnology Co., Ltd., Chengdu, China) was used as a reference. The standard curve is linear in the range of 0–0.07 mg/mL (Y = 27.758X−0.0245, R^2^ = 0.9918).

The epicatechin content was determined using high-performance liquid chromatography (HPLC) according to the Chinese Pharmacopeia (2020 edition). The materials were treated as follows: 6 g of *F. dibotrys* powder was precisely weighed, and 75 ml of an acetonitrile-0.2% phosphoric acid aqueous solution (9:1) was placed in a round-bottom flask. Heating reflux for 1 h. After heating reflux, the solution was collected, filtered, replenished, and purified through a filter syringe (0.45 μm). There were three replicates per sample. Epicatechin content was measured using HPLC using a Waters Alliance e2695 (Waters, WATERS Co., Ltd., MA, USA) and a Waters SunFire C18 column (4.6 mm × 250 mm, 5 um; WATERS Co., Ltd., MA, USA). Acetonitrile-0.2% phosphoric acid aqueous solution was used as the mobile phase, with gradient elution, a flow rate of 1 ml/min, a sample size of 10 μL, and the temperature of the column compartment maintained at 35°C. The epicatechin content of the sample is determined by the standard curve generated by the epicatechin standard (purchased from Chengdu Desite Biotechnology Co. Ltd., Chengdu, China). The standard curve is linear in the range of 0.05–0.25 μg/mL (Y = 831946X−14906, R^2^ = 0.9993).

### DNA extraction, amplicon, and high-throughput sequencing

Total genomic DNA was extracted from 0.5 g of soil and rhizome samples using the EZNA soil DNA kit (D5625-01, Omega Bio-tek Co., Ltd., Norcross, GA, USA) and the SQ Plant DNA Kit (D3095-01, Omega Bio-tek Co., Ltd., Norcross, GA, USA), according to the manufacturer's instructions. DNA quality was determined using the DeNovix DS-11 spectrophotometer (DeNovix Scientific, USA) the 1% agarose gel electrophoresis was used for qualitative judgment of DNA quality.

### PCR amplification of rhizome samples

The ITS sequence of endophytic fungi in rhizome samples was performed using nested PCR as previously described (Yao et al., 2019). The first round of PCR amplification was carried out using specific primers: ITS1F (5'-CTTGGTCATTTAGAGGAAGTAA-3') and ITS4 (5'-TCCTCCGCTTATTGATATGC-3') with the following thermal cycling program: 95 °C /5 min (initial denaturation), followed by 20 cycles at 94°C/1 min, 50 °C/50 s, 68°C/1 min, and finally at 68 °C/10 min (final extension). The second round of the PCR reaction used the diluted first-round PCR reaction product as a template and used primers: fITS7 (5'-GTGARTCATCGAATCTTTG-3') and ITS4 (5'-TCCTCCGCTTATTGATATGC-3') containing barcodes. The PCR thermal cycling program was as follows: 98°C for 1 min, followed by 19 cycles at 94°C/10 s, 50°C/30 s, and 72°C/45 s, and a final extension at 72°C/10 min. Each round PCR mixture (25 μL) contained 50 ng of DNA template, 2.5 μL of forward and reversed primers (1 μM for each), 12.5 μL of Phusion Hot Start Flex 2× Master Mix (Biolabs, New England), and 6.5 μL of distilled water. The PCR reaction program was as follows: 98°C for 30 s, followed by 32 cycles at 94°C for 10 s, 50°C for 30 s, and 72°C for 45 s, and a final extension at 72°C for 5 min.

### PCR amplification of soil samples from the rhizosphere

The ITS2 region of the rRNA genes of the rhizosphere soil samples was amplified as previously described (Karlsson et al., 2014). PCR amplification was carried out using primers: ITS1F (5'-GTGARTCATCGAATCTTTG-3') and ITS2 (5'-TCCTCCGCTTATTGATATGC-3'). The PCR reaction mix (25 μL) contained 50 ng DNA template, 2.5 μL of forward and reversed primers (1 μM for each), 12.5 μL of Phusion Hot Start Flex 2× Master Mix (Biolabs, New England), and 6.5 μL of distilled water. The PCR reaction program was a thermal cycling program: 98°C for 30 s, followed by 32 cycles at 98°C for 10 s, 54°C for 30 s, and 72°C for 45 s, and a final extension at 72°C for 10 min.

### Purification of PCR products and illumina sequencing

The PCR products were confirmed with 2% agarose gel electrophoresis, purified with AMPure XT beads (Beckman Coulter Genomics, Danvers, MA, USA), and quantified using the Qubit instrument (Invitrogen, USA). Amplicon pools were prepared for sequencing, and the quantity and size of the amplicon library were assessed with the Illumina Library Quantification Kit (Kapa Biosciences, Woburn, MA, USA) and an Agilent 2100 Bioanalyzer (Agilent, USA), respectively. According to the manufacturer's recommendations, the libraries were sequenced on the NovaSeq P6000 platform at LC-Bio Technology (LC-Bio Co., Ltd., Hang Zhou, China).

### Bioinformatics analysis and statistical analysis

Barcode and primer sequences were truncated from the reads. Then, the paired-end reads were assigned to samples based on their unique barcode and truncated by cutting off the barcode and primer sequence using Cutadapt software (https://cutadapt.readthedocs.io/en/stable/). Raw reads were filtered using fqtrim (v0.94) to obtain high-quality clean tags (Bokulich et al., 2013). The clean sequences were filtered using Vsearch (v2.3.4). After dereplicating using DADA2, amplicon sequence variants (ASVs) were obtained and assigned to a taxon. Alpha diversity and beta diversity were calculated using QIIME2 and represented using R software (v2.15.3) (Bolyen et al., 2019).

## Results

### Analysis of secondary metabolites in the rhizome of *F. dibotrys*

**Figure 2:**
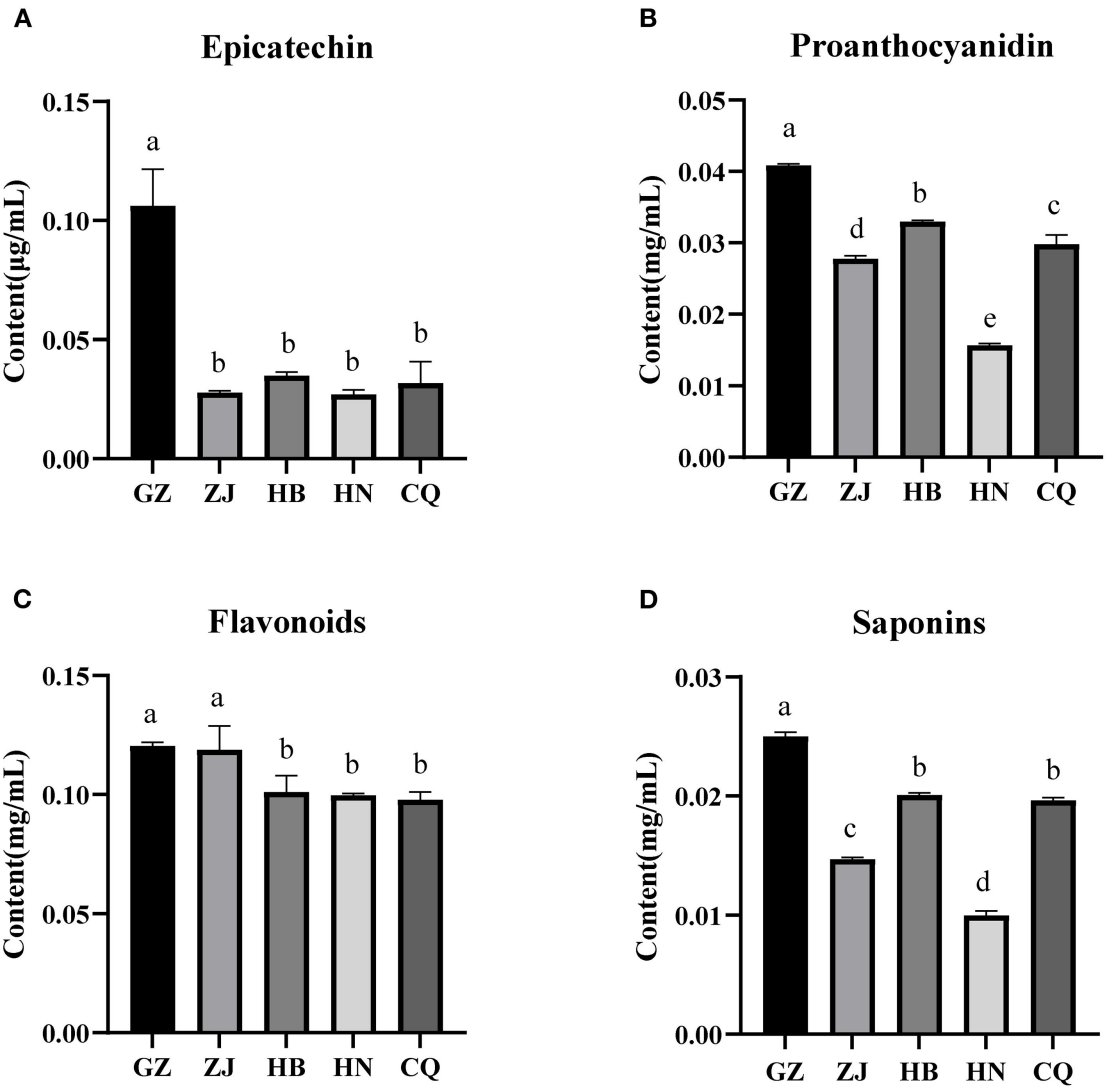
Secondary metabolite content in the rhizome of *F. dibotrys* from five areas. Epicatechin **(A)**, Proanthocyandidin **(B)**, Flavonoid **(C)**, Saponins **(D)**. The bar charts (mean with standard error) with different lowercase letters representing a significant difference (*P* < 0.05) were assessed by one-way ANOVA and followed by Duncan's multiple range test. CQ, HN, GZ, ZJ, and HB represent rhizome samples from Chongqing City, Hunan Province, Guizhou Province, Zhejiang Province, and Hubei Province, respectively.

The content of key secondary metabolites in the rhizome of *F. dibotrys* differed significantly in five areas. The epicatechin content in the GZ samples was significantly higher than that of the other four samples (*P* < 0.05), and there was no significant difference between ZJ, HB, HN, and QC (Figure 2A). There were significant differences in the proanthocyanin content between rhizome samples from five regions (*P* < 0.05), with GZ having the highest content, followed by HB, CQ, ZJ, and HN (Figure 2B). The total flavonoid content of the GZ and ZJ samples was significantly higher than that of the HB, HN, and CQ samples (*P* < 0.05) (Figure 2C). The total saponin content in the GZ samples was significantly higher than that of other areas (*P* < 0.05), while the HN samples exhibited a significantly lower saponin content than that of the other samples (*P* < 0.05) (Figure 2D). The results indicate that there are significant differences in the content of secondary metabolites in the rhizomes of different ecological regions. Among them, the GZ sample has a relatively higher secondary metabolite content, whereas the HN sample has a relatively lower content.

### Analysis of physicochemical properties in the rhizosphere soil

There were significant differences in the physicochemical properties of the rhizosphere soil across different regions. The pH values of soils showed that GZ, HB, and CQ had neutral soil conditions, HN exhibited weakly acidic soil characteristics, and ZJ had acidic soil conditions. The highest levels of total nitrogen (STN) and available nitrogen (SAN) were found in HB, followed by CQ, ZJ, and GZ, while HN displayed the lowest levels. The soil phosphorus content (STP) in the rhizosphere was highest in ZJ, followed by CQ, GZ, and HN, and lowest in HB. However, the SAP content was highest in ZJ and lowest in HN. The STK and SAK content in the rhizosphere soil is highest in CQ, followed by HB, HN, and ZJ, and lowest in GZ and CQ. Interestingly, the SAK content in the rhizosphere soil was highest in CQ and lowest in HN. Furthermore, the organic matter (OM) content in the rhizosphere soil was highest in CQ, followed by HB, ZJ, and GZ, while HN exhibited the lowest levels. The results indicated that the nutritional status of the HN rhizosphere soil was relatively poor (Table 1).

**Table 1:** The soil physical and chemical properties of *F. dibotrys* from different areas.

	**CQ**	**HN**	**GZ**	**ZJ**	**HB**
pH	6.78 ± 0.11b	6.17 ± 0.07c	7.35 ± 0.05a	5.6 ± 0.15d	7.17 ± 0.1a
STN(g/kg)	3.72 ± 0.16b	1.21 ± 0.1d	3.16 ± 0.02c	3.26 ± 0.02c	4.17 ± 0.02a
STP(g/kg)	0.8 ± 0.07b	0.49 ± 0.01c	0.79 ± 0.07b	1.28 ± 0.02a	0.42 ± 0.01c
STK(g/kg)	41.92 ± 1.91a	17.44 ± 0.87c	13.92 ± 0.81d	14.07 ± 0.72d	27.1 ± 0.13b
SAN(mg/kg)	383.95 ± 15.3a	155.28 ± 20.04c	328.92 ± 15.54b	348.45 ± 4.32b	351.81 ± 4.21b
SAP(mg/kg)	71.06 ± 1.45b	21.71 ± 1.75e	60.29 ± 3.39c	167.14 ± 4.73a	47.51 ± 1.43d
SAK(mg/kg)	832.12 ± 22.38a	202.73 ± 10.43e	248.61 ± 11.59d	286.96 ± 11.55c	459.63 ± 22.3b
OM (g/kg)	75.18 ± 2.27a	23.83 ± 2.01e	58.67 ± 2.71d	64.78 ± 0.62c	69.77 ± 0.97b

STN, soil total nitrogen; STP, Soil total phosphorus; STK, Soil total potassium; SAN, soil available nitrogen; SAP, soil available phosphorus; SAK, soil available potassium; OM, organic matter. Means are averages ± SD (n = 3). Different lowercase letters representing a significant difference (P < 0.05) were assessed by one-way ANOVA followed by Duncan's multiple range test.

**Figure 3:**
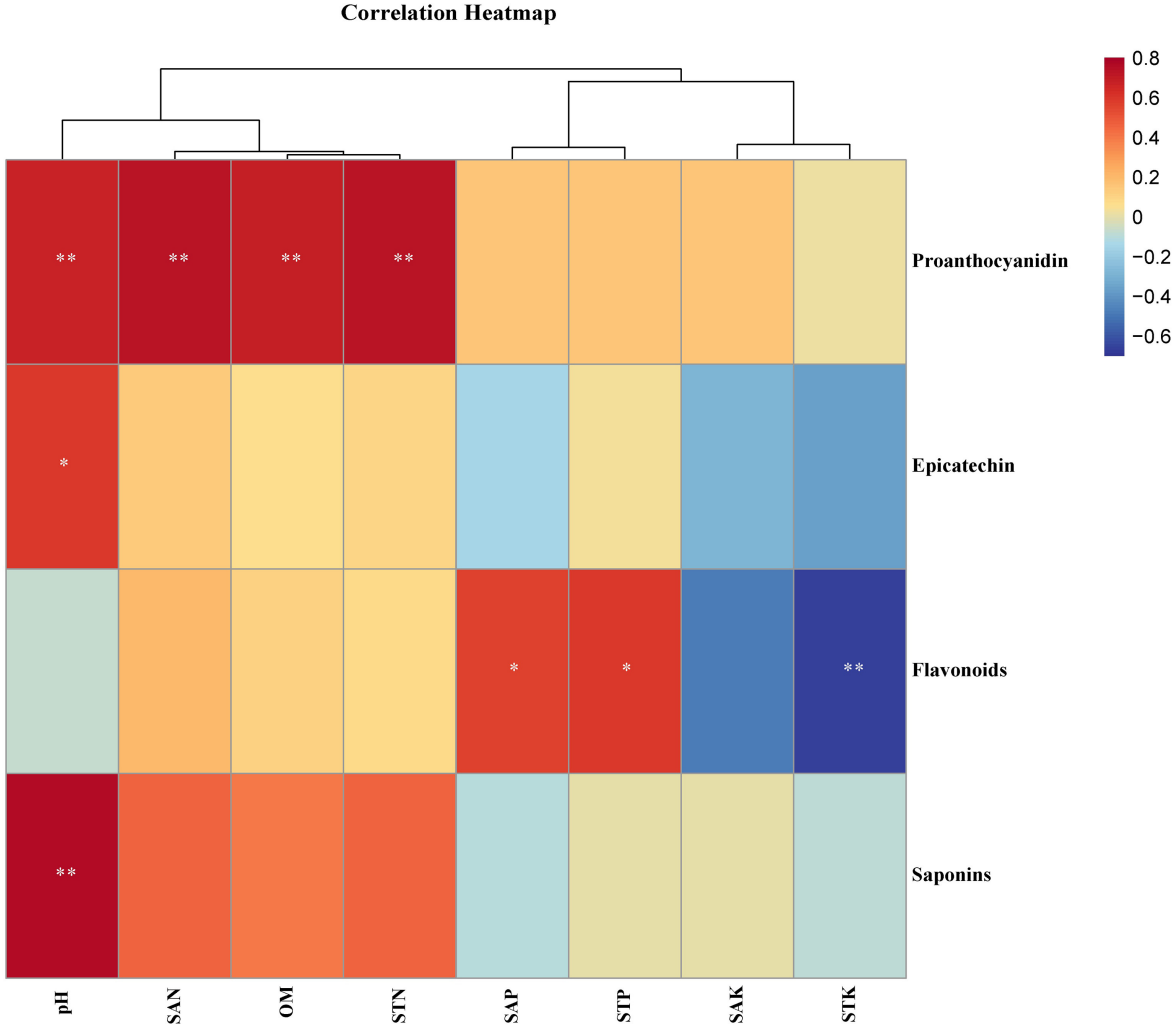
Pearson's correlation analysis between the content of secondary metabolites and soil physicochemical factors. **Indicates that the differences are significant at *P* < 0.01, and *indicates that the differences are significant at *P* < 0.05. CQ, HN, GZ, ZJ, and HB represent samples from Chongqing City, Hunan Province, Guizhou Province, Zhejiang Province, and Hubei Province, respectively.

To investigate the impact of soil nutrients on the quality of *F. dibotrys*, a correlation analysis was performed to evaluate the relationship between soil physical and chemical factors and secondary metabolites. The heat map of the correlation showed that the contents of proanthocyanidins were significantly positively correlated with the pH value, STN, SAN, and OM of the soil (*P* < 0.01). The content of epicatechin and saponins showed a significant positive correlation (*P* < 0.05) and an extremely significant positive correlation (*P* < 0.05), respectively, with the pH value. Notably, the content of total flavonoids was significantly positively correlated with SAP and soil STP (*P* < 0.05), while it was significantly negatively correlated with soil STK (*P* < 0.01). Specifically, the content of proanthocyanidins was significantly positively correlated with soil pH, SAN, OM, and STN. The epicatechin content was significantly positively correlated with soil pH. The total flavonoid content was significantly positively correlated with soil SAP and STP and significantly negatively correlated with soil STK. Total saponin content was positively correlated with soil pH (*P* < 0.05) (Figure 3).

### Diversity of rhizome endophytic fungal and rhizosphere soil fungal communities

**Figure 4:**
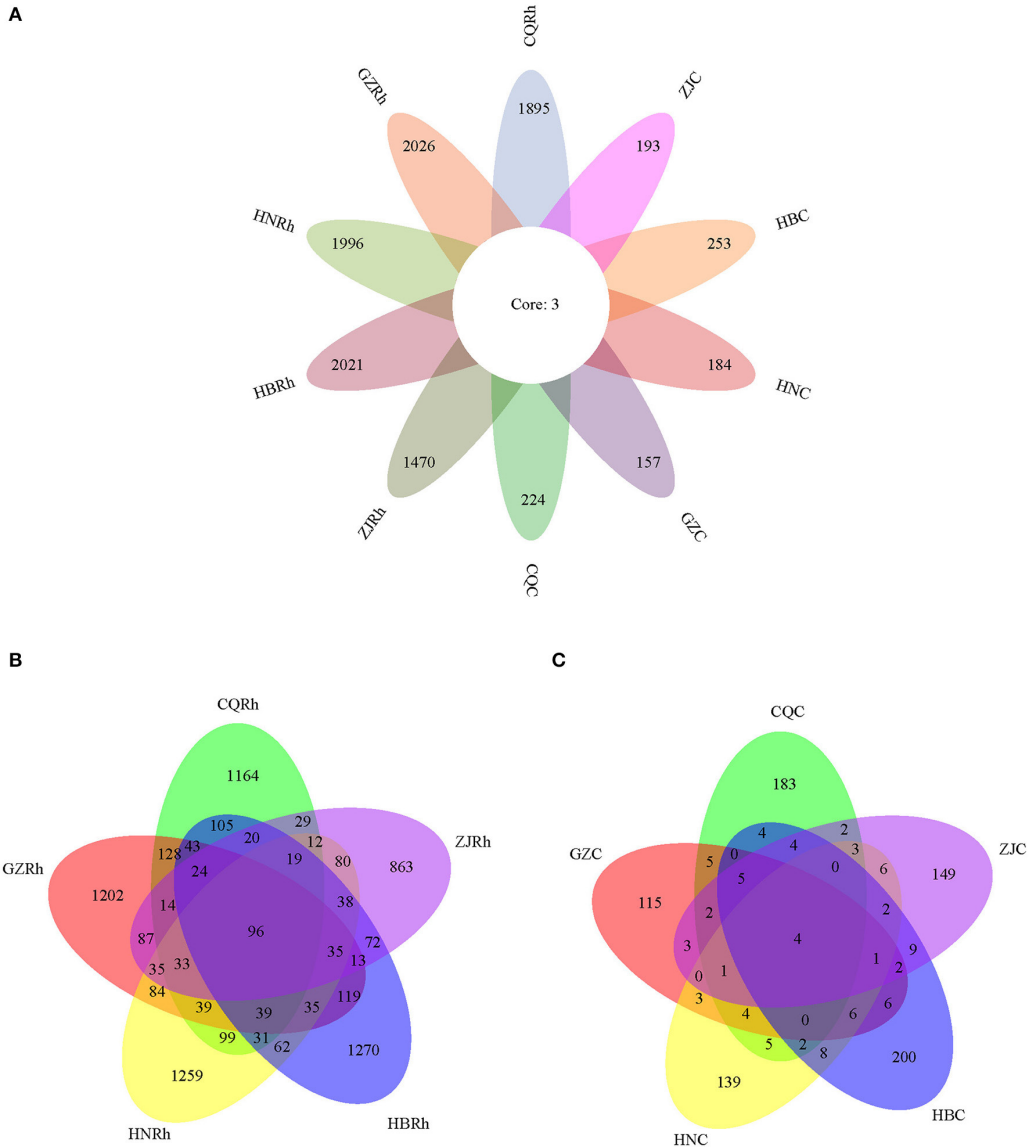
Distribution differences of endophytic fungi in the *F. dibotrys* sample from five different areas. The petal diagram **(A)** and the Venn diagram **(B, C)** are based on amplicon sequence variants (ASVs), representing common or unique ASVs for a given group. CQC, HNC, GZC, ZJC, and HBC represent endophytic fungal communities of rhizomes in *F. dibotrys* of Chongqing City, Hunan Province, Guizhou Province, Zhejiang Province, and Hubei Province, respectively. CQRh, HNRh, GZRh, ZJRh, and HBRh represent soil fungal communities from the rhizosphere in *F. dibotrys* of Chongqing City, Hunan Province, Guizhou Province, Zhejiang Province, and Hubei Province, respectively.

To determine the endophytic fungal community and the corresponding fungal community of rhizosphere soil in *F. dibotrys* from different ecological environments, we extracted DNA from the surface-sterilized rhizome of *F. dibotrys* and rhizosphere soil. Then, we determined the fungal community using high-throughput sequencing technology. A total of 10,422 ASVs were obtained from five rhizosphere soil groups and five rhizome groups. The ASVs of the fungi in the rhizosphere soil were found to be much higher than those of the endophytic fungi in the rhizome. The highest number of fungal ASVs in the rhizosphere soil was 2,029 in GZ, followed by 2,024 in HB, 1,999 in HN, 1,899 in CQ, and the minimum number was 1,473 in ZJ. Regarding the endophytic fungal community, the highest number of fungal ASVs was 256 in HB, followed by 227 in CQ, 200 in ZJ, and 187 in HN. The minimum number was 160 in GZ. Among the groups of rhizome and rhizosphere soil from five different regions, there were only three shared fungal ASVs, all of which belonged to Ascomycota, of which two ASVs belonged to the *Cylindrocarpon* genus and one ASV belonged to the *Bionectria* genus (Figure 4A). As shown in the petal diagram, four shared fungal ASVs were presented in the fungal community of five rhizome samples, and 96 shared fungal ASVs were presented in the fungal community of five rhizosphere soil samples (Figures 4B, C). The results showed that the diversity of rhizosphere soil fungal communities was much greater than that of endophytic fungi, and the diversity of soil fungal communities showed significant differentiation due to different regional ecological environments.

The alpha diversity indices (Shannon, Simpson, Chao1, and ACE index) of endophytic fungal and rhizosphere soil fungal communities between different groups were performed using the Wilcoxon rank-sum test. The results showed that the richness (Chao1 and ACE index) of the rhizosphere soil fungal communities and endophytic fungal communities did not show any significant differences between the groups. However, the diversity (Shannon and Simpson index) of rhizosphere soil fungal communities and endophytic fungal communities was significantly different between the different groups (*P* < 0.05). The Shannon index of endophytic fungal communities in HBC and GZC samples was significantly higher than that of ZJC samples, while the Simpson index of endophytic fungal communities in ZJC samples was significantly lower than that of the remaining four other samples (*P* < 0.05). Furthermore, the Shannon and Simpson index of the fungal communities of the rhizosphere soil in HBRh was significantly lower than that of the remaining four other samples (*P* < 0.05) (Table 2). It showed that the different ecological environments significantly affected the diversity rather than the richness of the endophytic and rhizosphere soil fungal communities in *F. dibotrys*.

**Table 2:** Alpha diversity indices of endophytic fungal community and rhizosphere soil fungal community in *F. dibotrys*.

**Sample**	**Endophytic fungi**				**Rhizosphere soil fungi**			
	**Shannon**	**Simpson**	**chao1**	**ace**	**Shannon**	**Simpson**	**chao1**	**ace**
CQ	2.78 ± 0.08ab	0.72 ± 0.025a	92.62 ± 28.58	90 ± 28.14	7.02 ± 0.14a	0.98 ± 0.002a	971.6 ± 66.15	967.88 ± 79.07
GZ	4.05 ± 0.74a	0.88 ± 0.054a	76.67 ± 32.35	77.63 ± 33.27	7.29 ± 0.05a	0.98 ± 0.006a	943.53 ± 63.48	975.77 ± 29.62
HB	3.67 ± 1.5a	0.73 ± 0.277a	108.06 ± 25.53	110.15 ± 22.77	6.5 ± 0.22b	0.94 ± 0.009b	951.56 ± 39.34	965.3 ± 90.63
HN	2.98 ± 0.54ab	0.81 ± 0.067a	78.87 ± 15.7	79.36 ± 17.087	7.37 ± 0.35a	0.98 ± 0.006a	962.5 ± 243.19	973.86 ± 246.33
ZJ	1.56 ± 0.45b	0.39 ± 0.16b	85.42 ± 30.92	84.2 ± 28.51	7.27 ± 0.21a	0.98 ± 0.009a	795.89 ± 28.2	798.74 ± 15.3

Means are averages ± SD (n = 3). Different lowercase letters representing a significant difference (P < 0.05) were assessed by one-way ANOVA followed by Duncan's multiple range test.

**Figure 5:**
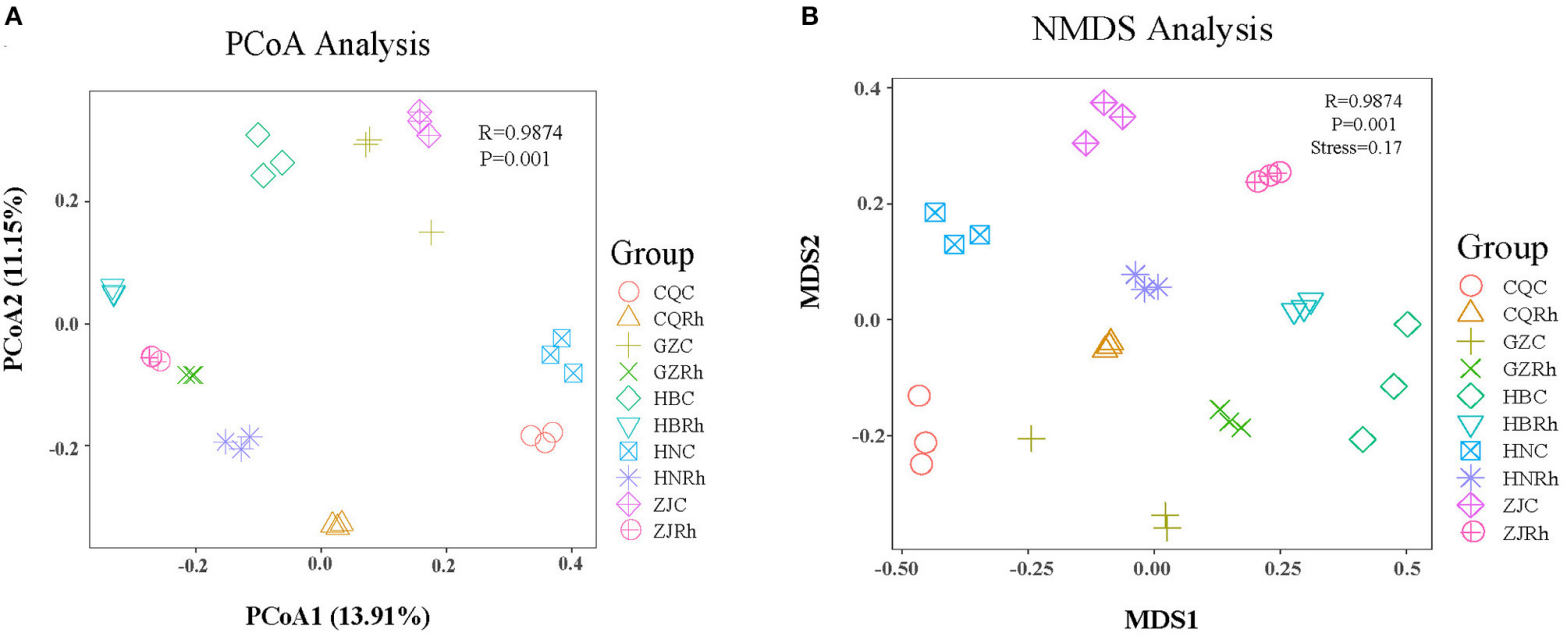
Beta diversity analysis of fungal communities based on unweighted UniFrac distance. Principal Coordinate Analysis (PCoA) **(A, B)** Nonmetric Multidimensional Scaling (NMDS) analysis of rhizosphere soil fungal and endophytic fungal communities.

The principal coordinate analysis (PCoA) based on the Bray–Curtis distance algorithm was also conducted to reveal the differences between different rhizosphere soils and endophytic fungi of the rhizome in *F. dibotrys*. The PCoA result showed that soil samples from the rhizosphere (CQRh, ZJRh, GZRh, HNRh, HBRh) and the rhizome (CQC, ZJC, GZC, HNC, HBC) were clearly separated (Figure 5A). Nonmetric multidimensional scale (NMDS) analysis showed that fungal communities in rhizome and rhizosphere soil from five different ecological regions were clearly isolated. Furthermore, the stress analyzed by NMDS was 0.17, proving the statistical method's precision (Figure 5B). The results demonstrated that different ecological regions had significant effects on the structure and diversity of the community of rhizosphere soil and endophytic fungi in *F. dibotrys*.

### Composition of endophytic fungal and rhizosphere soil fungal communities

**Figure 6:**
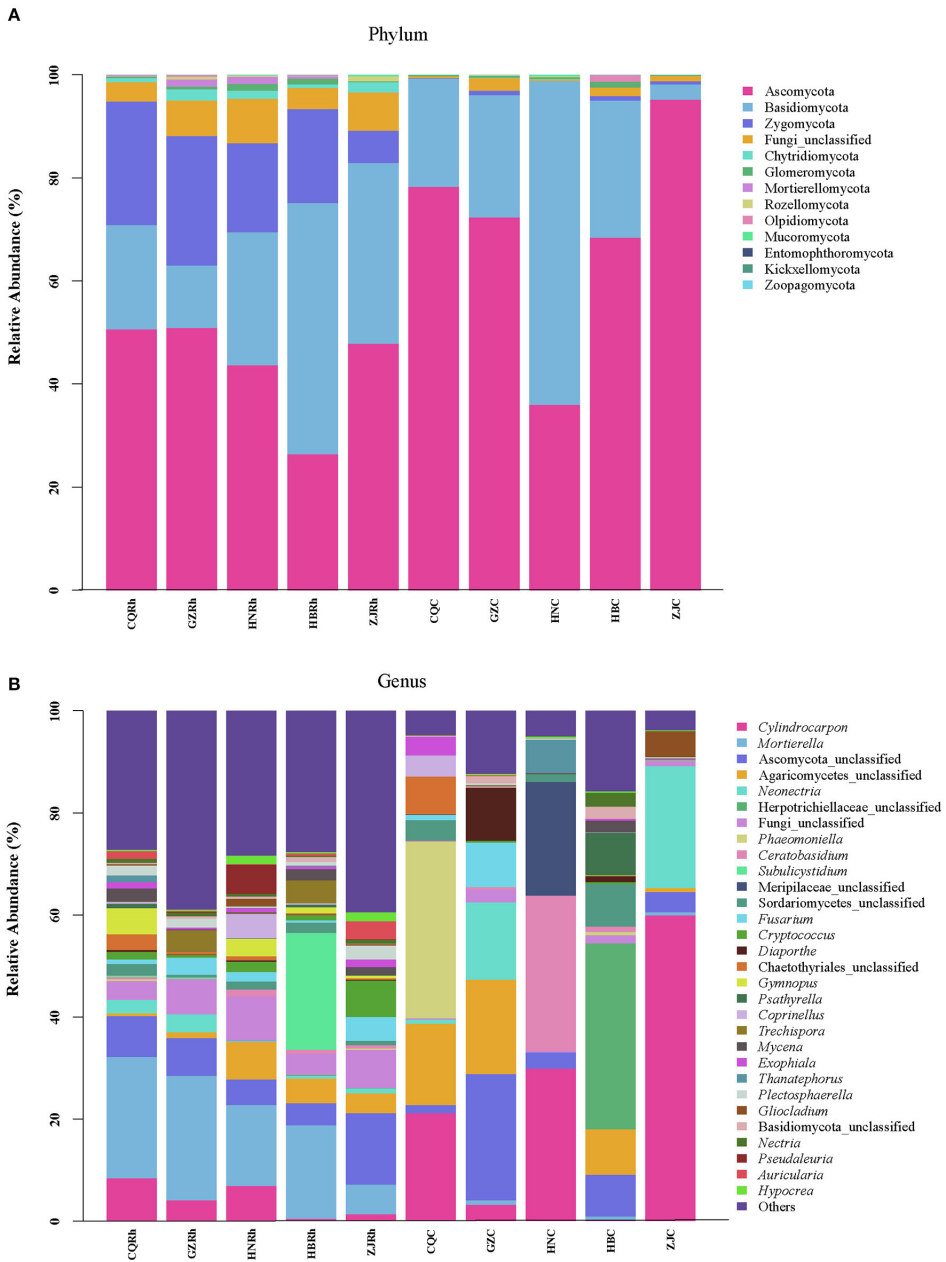
Composition of the top 30 endophytic and rhizosphere soil fungi in *F. dibotrys* from different areas **(A)** Phylum level, **(B)** genus level.

According to species abundance and annotation results, ASVs were assigned 13 phyla, 60 classes, 147 orders, 317 families, and 663 genera, respectively. At the phylum level, Ascomycota was the dominant phylum in all samples, with relative abundances ranging from 36.01% to 68.45%, except for the fact that Basidiomycota was dominant in HBRh (48.79%) and HNC (62.85%) (Figure 6A). At the genus level, *Mortierella* was the dominant genus in three samples (CQRh, GZRh, and HNRh), with relative abundances of 23.81%, 24.36%, and 15.91%, respectively. *Subulicystidium* and Ascomycota_unclassified were the dominant genus in the HBRh sample (22.94%) and ZJRh (14.00%), respectively. *Cylindrocarpon* was the dominant genus in the HNC sample (29.87%) and the ZJC sample (59.93%). Furthermore, Ascomycota_unclassified, Herpotrichiellaceae_unclassified, and *Phaeomoniella* were the dominant genus in the GZC (24.75%), HBC sample (36.44%), and CQC sample (34.63%), respectively (Figure 6B). The results suggested greater differences in the composition and structure of endophytic fungal communities in the rhizome compared to rhizosphere soil fungal communities in different ecological environments.

### Relationship of rhizome secondary metabolites with soil physicochemical properties and fungal community

**Figure 7:**
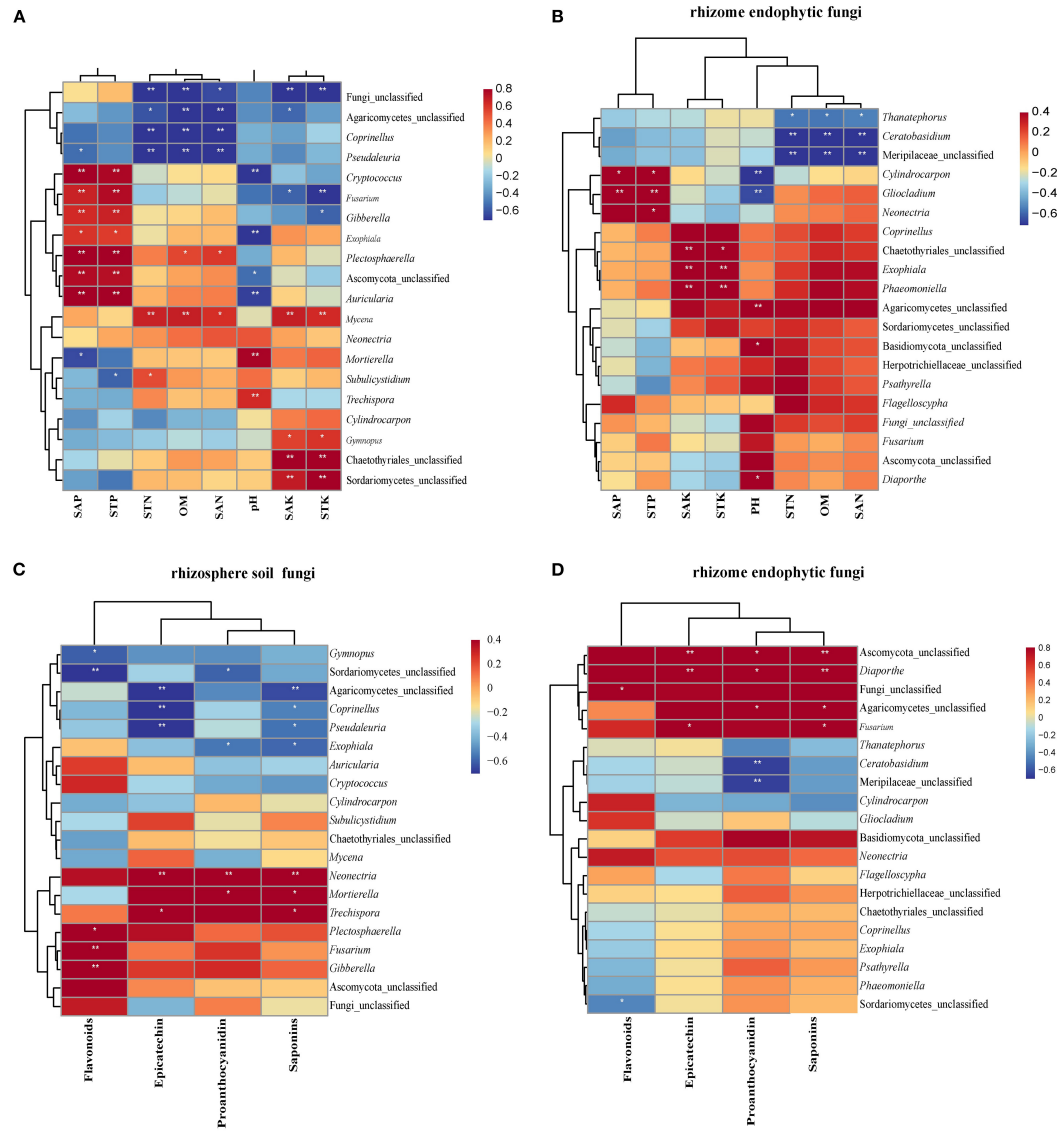
Relationship between secondary metabolites, soil physicochemical factors, and fungal communities. Heat maps of Pearson's correlation analysis between soil physicochemical factors and the top 20 ASVs at the genus level in endophytic fungal **(A)** and rhizosphere soil fungal communities **(B)**, respectively. Heat maps of Pearson's correlation analysis between secondary metabolites and the top 20 ASVs at the genus level in endophytic fungal communities **(C)** and rhizosphere soil fungal communities **(D)**, respectively. The label list in the Figures indicates the value of the correlation coefficient. **indicates that the differences are significant at *P* < 0.01, and *indicates that the differences are significant at *P* < 0.05.

The correlation analysis between the TOP20 genera of ASVs in rhizosphere soil fungal communities and soil physicochemical properties is depicted using the Pearson correlation heat map. Specifically, *Cryptococcus, Fusarium, Gibberella, Plectosphaerella*, and Ascomycota_unclassified, *Auricularia* had a positive correlation with SAP and STP; *Pseudaleuria* and *Mortierella* were negatively correlated with SAP; and *Subulicystidium* was negatively correlated with STP. Furthermore, Fungi_unclassified, Agaricomycetes_unclassified, *Coprinellus*, and *Pseudaleuria* were negative, while *Mycena* was positive and correlated with STN, OM, and SAN, respectively. Furthermore, *Plectosphaerella* was positively correlated with OM and SAN; and *Subulicystidium* was positively correlated with STN. *Cryptococcus, Exophiala*, Ascomycota_unclassified, and *Auricularia* were negatively correlated with pH value, while *Mortierella* and *Trechispora* were positively correlated with pH value. Fungi_unclassified, Agaricomycetes_unclassified, and *Fusarium* were negatively correlated, while *Mycena, Gymnopus*, Chaetothyriales_unclassified, and Sordariomycetes_unclassified were positively correlated with SAK. Furthermore, Fungi_unclassified, *Fusarium* and *Gibberella* were negatively correlated, while *Mycena, Gymnopus*, Chaetothyriales_unclassified, and Sordariomycetes_unclassified were positively correlated with STK (Figure 7A).

In terms of correlation between TOP20 ASV genera in endophytic fungal communities of the rhizome and physicochemical properties of soil from the rhizosphere, *Cylindrocarpon* and *Gliocladium* were positively correlated with SAP and STP, and *Neonectria* was positively STP. *Coprinellus*, Chaetothyriales_unclassified, *Exophiala*, and *Phaeomoniella* were positively correlated with pH value, while Agaricomycetes_unclassified, Basidiomycota_unclassified, and *Diaporthe* were negative. Notably, *Thanatephorus, Ceratobasidium*, and Meripilaceae_unclassified showed a significant negative correlation with STN, OM, and SAN (Figure 7B).

According to the Pearson correlation heat map between the TOP20 genera of rhizosphere soil fungal communities and the secondary metabolites in the rhizome, *Gymnopus*, and Sordariomycetes_unclassified were positively correlated, while *Plectosphaerella, Fusarium*, and *Gibberella* were negatively correlated with flavonoids. Agaricomycetes_unclassified, *Coprinellus*, and *Pseudaleuria* were negatively correlated, while *Neonectria* and *Trechispora* were positively correlated with epicatechin. Sordariomycetes_unclassified and *Exophiala* were negatively correlated, while *Neonectria* and *Mortierella* were positively correlated with proanthocyanidin. Moreover, Agaricomycetes_unclassified, *Coprinellus, Pseudaleuria*, and *Exophiala* were negatively correlated, while *Neonectria, Mortierella*, and *Trechispora* were positively correlated with saponins.

According to the Pearson correlation heat map between the TOP20 genera of endophytic fungal communities of the rhizome and the secondary metabolites in the tuber root, Fungi_unclassified was positively correlated with flavonoids (Figure 7C). Ascomycota_unclassified, *Diaporthe*, and *Fusarium* were positively correlated with epicatechin. Ascomycota_unclassified, *Diaporthe*, and Agaricomycetes_unclassified were positively correlated, while *Ceratobasidium* and Meripilaceae_unclassified were negatively correlated with anthocyanin. Furthermore, proanthocyanidin was positively correlated with Ascomycota_unclassified, *Diaporthe*, Agaricomycetes_unclassified, and *Fusarium* (Figure 7D).

**Figure 8:**
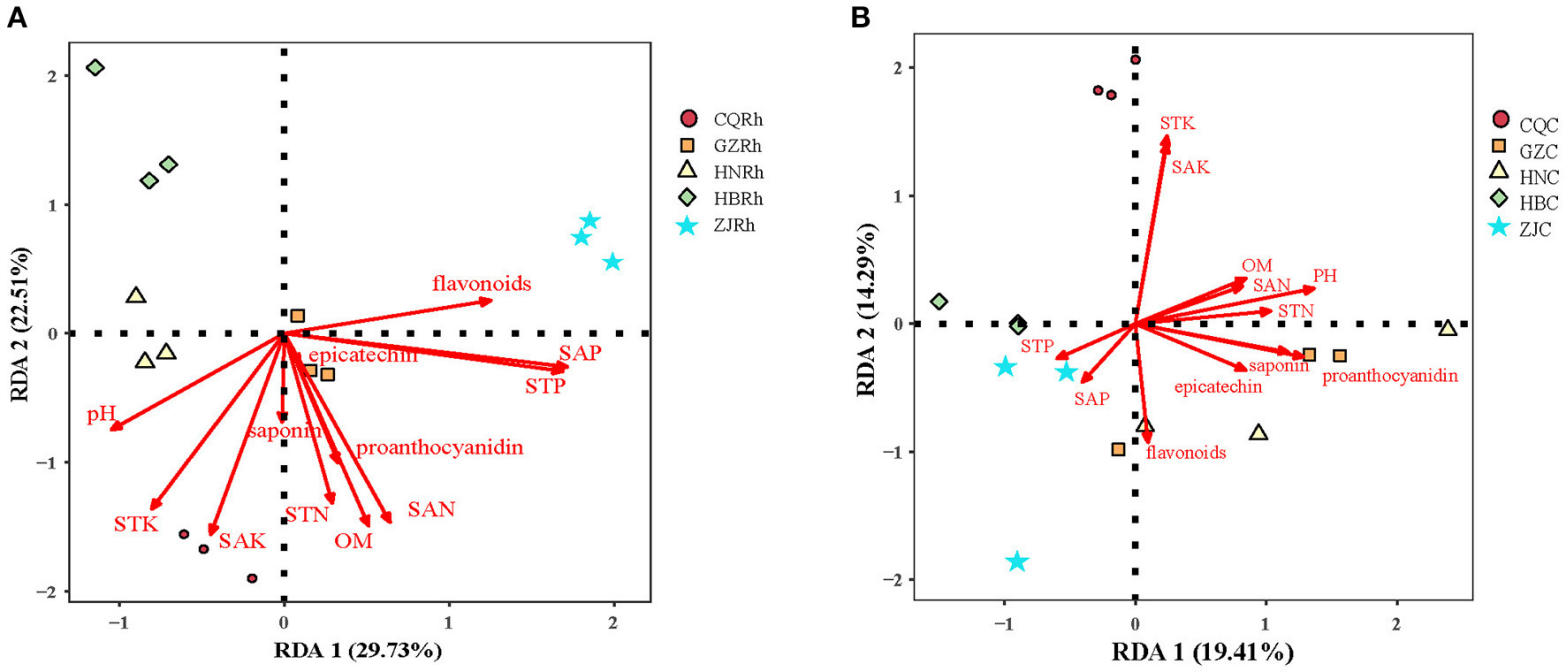
Redundancy discriminant analysis (RDA) analysis of the relationship between fungal communities and soil physicochemical factors. Endophytic fungal communities **(A)** and rhizosphere soil fungal communities **(B)**, respectively.

Redundancy analysis (RDA) was conducted to further determine the effects of soil physicochemical factors on soil fungal communities in the rhizosphere and endophytic fungal communities in the rhizome. In the rhizome endophytic fungal community, the first two RDA axes explained 19.41% and 14.21% of the total variance, respectively, for a total of 33.62%. Soil pH (*r*^2^ = 0.597, *P* < 0.01), STK (*r*^2^ = 0.660, *P* < 0.01), SAK (*r*^2^ = 0.610, *P* < 0.01), saponins (*r*^2^ = 0.431, *P* < 0.05) and proanthocyanidin (*r*^2^ = 0.522, *P* < 0.05) were crucial environmental factors that affected the distribution of fungal communities in the rhizosphere soil (Figure 8A). Regarding the endophytic fungal communities of the rhizome, the first two axes of the fungal RDA in the rhizosphere soil explained 29.55% and 21.73% of the total variance, respectively, accounting for a total of 51.66%. Soil pH (*r*^2^ = 0.512, *P* < 0.05), STK (*r*^2^ = 0.770, *P* < 0.01), SAK (*r*^2^ = 0.808, *P* < 0.01), STN (*r*^2^ = 0.554, *P* < 0.01), OM (*r*^2^ = 0.758, *P* < 0.01), SAN (*r*^2^ = 0.780, *P* < 0.01), STP (*r*^2^ = 0.902, *P* < 0.01), and SAP (*r*^2^ = 0.925, *P* < 0.01) were crucial environmental driving factors that affected the distribution of endophytic fungal communities of rhizome (Figure 8B). The results indicated that the rhizosphere soil fungal communities are more sensitive to soil physicochemical factors and secondary metabolites than the endophytic fungal communities.

## Discussion

The interactions among plant–microbe–soil systems regulate soil ecosystems, plant growth and development, and biotic and abiotic stress responses (Upton et al., 2019). Secondary metabolites from medicinal plants maintain their concentration during the growth period and protect plants from the influence of neighboring plants, pathogens, and constantly changing environmental conditions (Zhang et al., 2021b). Secondary metabolism was affected by multiple internal and external factors, in which rhizosphere soil and endophytic fungal communities affecting plant secondary metabolism have received great attention (Li et al., 2020; Pang et al., 2021; Zhang et al., 2021b).

It was established that there are different dominant genera in each region. For example, *Mortierella* was the dominant genus in three samples (CQRh, GZRh, and HNRh) (Figure 6A). *Mortierella* is a common genus of beneficial agricultural fungi in the soil that is commonly used to improve nutrient absorption efficiency, protect crops from adverse conditions, and reduce the application of fertilizers and pesticides (Nguyen et al., 2019; Ozimek and Hanaka, 2021). The abundance of *Mortierella* is positively correlated with the pH value and negatively correlated with SAP (Figure 7A). In addition, it is positively correlated with Proanthocyanidin and total saponins (Figure 7C), indicating that *Mortierella* may be affected by soil pH and SAP and play an important role in plant secondary metabolism. *Cylindrocarpon* was the dominant genus in the HNC sample (29.87%) and the ZJC sample (59.93%) (Figure 6B). *Cylindrocarpon* is a parasitic soil fungus that occasionally acts as a human and animal pathogen (Song et al., 2014), hinting at the fact that the HNC sample may contain a certain number of pathogenic fungi. Furthermore, *Phaeomoniella* was the dominant genus in the CQC sample (34.63%) (Figure 6B). The abundance of *Phaeomoniella* is positively correlated with SAK and STK (Figure 6B). *Phaeomonella* is a common endophytic plant fungus. Fungal grapevine trunk diseases (GTDs) are some of the most pressing threats to grape production worldwide. These diseases are associated with several fungal pathogens, such as *Phaeomoniella chlamydospora* and *Phaeoacremonium minimum* (Chen et al., 2015; Ramsing et al., 2021). The results suggested that excessive potassium may increase the abundance of *Phaeomonella*, which provides important information for the ecological cultivation of *F. dibotrys*.

In this study, the relationships between the secondary metabolites of the rhizome and the physicochemical properties of the soil were examined in *F. dibotrys* from five areas. Our studies showed that the content of secondary metabolites, that is, epicatechin proanthocyanidins, total flavonoids, and total saponins, in the *F. dibotrys* rhizome showed significant differences between the five areas (Figures 2A–D), which is consistent with the previous study that the quality of *F. dibotrys* from different regions varies greatly (Zhang et al., 2022). It has been shown that the content of bioactive ingredients is significantly associated with soil physiochemical properties in root-associated medicinal parts (Dang et al., 2021; Miransari et al., 2021). Pearson's correlation analysis in our study revealed that the content of secondary metabolites in the rhizome was significantly associated with the soil physicochemical properties of *F. dibotrys* (Figure 3). In addition, the content of proanthocyanidins, epicatechin, and saponins was positively correlated with soil pH, and the content of proanthocyanidins was also positively correlated with SAN, STN, and OM.

Furthermore, the content of flavonoids was positively correlated with SAP and STP while negatively correlated with STK (Figure 3). It suggested that soil pH, SAN, STN, OM, SAP, STP, and STK played crucial roles in the accumulation of metabolites in the rhizome of *F. dibotrys*. As is known, N fertilizers are determinant nutrients for plant biomass or crop yield and are commonly applied in agricultural practice to increase crop yield (Khan et al., 2020). Moderate N fertilization was shown to improve the colonization of the bacterial and fungal community in rice seeds and can improve grain yield and quality, while overuse of N can lead to decreased grain eating and cooking quality (Liu et al., 2023). Previous studies have reported that the flavonoid content in licorice is significantly influenced by environmental factors, and the secondary metabolites (GIA, GTF, and LI) of licorice are significantly positively correlated with SAN (Xie et al., 2018; Dang et al., 2021). Cun et al. (2023) reported that excessive application of N improved root yield but reduced the accumulation of saponins in *Panax notoginseng* (Cun et al., 2023). The different results mentioned above suggested no excessive application of N, and the content of N is positively correlated with secondary metabolites in the wild environment. However, the application of N is prone to excessive use under cultivated conditions, resulting in a significant increase in yield and a decrease in the content of secondary metabolites. P can promote carbohydrate synthesis and transport, improve protein synthesis and fat metabolism, and play key roles in plant drought, cold, and disease resistance (Timofeeva et al., 2022). Chaouqi et al. (2023) revealed that P in the soil was conducive to the synthesis of secondary metabolites such as flavonoids. Potassium is the main nutrient necessary for plant growth and development and plays an important role in primary and secondary metabolites such as enzyme activation, protein synthesis, photosynthesis, and ion homeostasis (Hafsi et al., 2017; Lu et al., 2023). Our study showed that the content of flavonoids was negatively correlated with STK. Therefore, an appropriate amount of K nutrition is beneficial for plant growth and development, balancing the accumulation of secondary metabolites and the stress response in *F. dibotrys*.

Plants often modify their soil environment and harbor their ubiquitous soil microbiome by providing important habitats and providing photosynthates to them (Bennett et al., 2017; Jiang et al., 2017). In turn, the growth and development processes of plants are also determined by the soil-associated microbiome, which plays an essential role in the availability of nutrients, ecological functions, and secondary metabolite production in plants (Lau and Lennon, 2011; Bennett et al., 2017; Jiao et al., 2019). The temporal dynamics of microbial communities, including root-related microbiomes, are influenced by many factors, such as soil pH, nitrogen, and phosphorus content (Edwards et al., 2015). Many studies have shown a high correlation between the accumulation of secondary metabolites in medicinal plant soil fungal communities and the rhizosphere, such as *P. ginseng* (Wei et al., 2020), *Glycyrrhiza uralensis* (Liu et al., 2023); *Glehnia littoralis* (Liu et al., 2023); and endophytic fungal communities, such as licorice (He et al., 2019; Dang et al., 2021), *Rheum palmatum* (Chen et al., 2021), *Sophora alopecuroides* (Ju et al., 2022), and *Huperzia serrata* (Pang et al., 2022). In this study, HTS was carried out to estimate the composition and diversity of endophytic fungal and rhizosphere soil fungal communities in *F. dibotrys* and elucidate the role of fungal communities in secondary metabolites in *F. dibotrys*. Based on our results, the diversity (Shannon and Simpson indices) of endophytic fungi and rhizosphere soil fungal communities showed significant differences between different groups (*P* < 0.05), while the richness of them (Chao1 and ACE indices) did not show any significant differences (Table 2). The results revealed that different ecological environments of *F. dibotrys* significantly affect the diversity of endophytic fungi and rhizosphere soil fungal communities rather than their richness. Only four shared fungal ASVs were identified in five rhizome samples, and 96 shared fungal ASVs were identified in five rhizosphere soil samples, respectively (Figures 4A–C). The large number of unique fungal communities present in rhizomes and rhizosphere soil was determined by the unique internal environment and the ecological habitat environment of *F. dibotrys*, which are the result of the co-evolution of plants and microorganisms (Albrectsen et al., 2018; Upton et al., 2019). Furthermore, we found that a large number of endophytic fungi and rhizosphere soil fungi in *F. dibotrys* could not be classified. The fact is that only approximately 10,000 out of approximately 5.1 million fungal species have been classified and identified so far (Blackwell, 2011). Therefore, HTS is more capable of comprehensively exploring the structural composition of endophytic fungal and rhizosphere soil communities compared to traditional colony separation methods (Siddique et al., 2017).

It is known that plant-derived compounds (i.e., flavonoids, saponins, etc.) have diverse biological functions in plants, and flavonoids can act as signaling components in regulating plant-microbe symbioses in the rhizosphere (Hassan and Mathesius, 2012). Furthermore, many flavonoids have been reported to have multiple functions as irritants in one fungus and inhibitors in another fungus, dynamically affecting the abundance of different endophytic fungi (Hassan and Mathesius, 2012). According to the outcome of our study, the numbers of endophytic fungi and rhizosphere soil fungi were positively or negatively correlated with the secondary metabolites of *F. dibotrys*. For example, *Gymnopus* and Sordariomycetes_unclassified in rhizosphere soil were positively correlated, while *Plectosphaerella, Fusarium*, and *Gibberella* were negatively correlated with flavonoids (Figure 8). Similar results were found in other medicinal plants, such as *Cynomorium songaricum* (Cui et al., 2018), *R. palmatum* (Chen et al., 2021), *Ginkgo biloba* (Wu et al., 2022), *Polygonum hydropiper*, and *P. lapathifolium* (Zhang et al., 2022). Furthermore, the correlation between secondary metabolites of *F. dibotrys* and endophytic fungi in the rhizome and rhizosphere soil fungi was significantly different (Figures 7C, D), which revealed that endophytic fungi and rhizosphere soil fungi could interact with secondary metabolites in a different manner. Additionally, some rhizosphere soil fungi may directly or indirectly affect the medicinal value of *F. dibotrys*. Studies have shown that the soil fungus *Mycena* can synthesize alkaloids (Jaeger et al., 2013). *Pseudaleuria* promoted the accumulation of fungal residues and was beneficial to the storage of soil C (Chen et al., 2022). *Mortierella* can transform insoluble P in soil into soluble P, improve plant absorption of P, and thus promote plant growth and development (Sang et al., 2022). Interestingly, *Diaporthe* in the rhizosphere soil was found to be significantly positively correlated with the content of epicatechin, proanthocyanidins, and total saponins (Figure 7C). Previous studies have reported that *Diaporthe* is widely found in many plants, and a variety of active compounds have been extracted from it, such as polyketones, terpenes, phenols, alkaloids, etc. (Xu et al., 2021; Gu et al., 2022). In the current study, the abundance of *Diaporthe* is highest in the rhizome of the GZ sample, and the quality of the rhizome of GZ is the best among the five samples. However, more research is needed to determine whether there is a direct relationship between the abundance of *Diaporthe* and the medicinal quality of *F. dibotrys*.

It should be noted that, owing to the complexity of the interaction between plants, soil, and microorganisms, determining their roles and the exact mechanism of their interaction under natural conditions is difficult. However, the newer technologies, such as next-generation and single-molecule real-time (SMRT) sequencing, are gradually enabling the exploration of the causal relationships between them. The current study provides valuable insights into understanding the interactions, roles, and microorganisms in *F. dibotrys*, contributing to the ecological cultivation and quality control of this species.

## Conclusion

The ITS region of endophytic fungi and rhizosphere soil fungi in *F. dibotrys* from five areas was sequenced based on high-throughput sequencing, and the physicochemical properties of rhizosphere soil and the key secondary metabolites of rhizomes were also measured. The results showed that the soil pH, soil N, OM, and P were significantly correlated with the active components of *F. dibotrys*. Furthermore, different ecological environments of *F. dibotrys* significantly affect the diversity of endophytic fungi and rhizosphere soil fungal communities rather than their richness.

## Data availability statement

The datasets presented in this study can be found in online repositories. The names of the repository/repositories and accession number(s) can be found at: https://www.ncbi.nlm.nih.gov/genbank/, SRR24288003- SRR24288032.

## Author contributions

NM conducted the experiments, analyzed the data, and wrote the manuscript. DY, YL, ZG, YC, TC, and ZH collected samples and contributed to the experiments. QJ and DW supervised the experiments. DW wrote and critically reviewed the manuscript. All authors contributed to the article and approved its submitted version.
